# Comparative transcriptional analysis of *Candida auris* biofilms following farnesol and tyrosol treatment

**DOI:** 10.1128/spectrum.02278-23

**Published:** 2024-03-05

**Authors:** Ágnes Jakab, Fruzsina Kovács, Noémi Balla, Csaba Nagy-Köteles, Ágota Ragyák, Fruzsina Nagy, Andrew M. Borman, László Majoros, Renátó Kovács

**Affiliations:** 1 Department of Medical Microbiology, Faculty of Medicine, University of Debrecen, Debrecen, Hungary; 2 Doctoral School of Pharmaceutical Sciences, University of Debrecen, Debrecen, Hungary; 3 Department of Molecular Biotechnology and Microbiology, Institute of Biotechnology, Faculty of Science and Technology, University of Debrecen, Debrecen, Hungary; 4 Department of Inorganic and Analytical Chemistry, Agilent Atomic Spectroscopy Partner Laboratory, University of Debrecen, Debrecen, Hungary; 5 UK National Mycology Reference Laboratory, UK Health Security Agency, Science Quarter, Southmead Hospital, Bristol, United Kingdom; 6 Medical Research Council Centre for Medical Mycology (MRCCMM), University of Exeter, Exeter, United Kingdom; Universidade de Brasilia, Brasília, Brazil

**Keywords:** *Candida auris*, transcriptome, quorum-sensing, ergosterol, calcium, magnesium, iron, biofilm

## Abstract

*Candida auris* is frequently associated with biofilm-related invasive infections. The resistant profile of these biofilms necessitates innovative therapeutic options, where quorum sensing may be a potential target. Farnesol and tyrosol are two fungal quorum-sensing molecules with antifungal effects at supraphysiological concentrations. Here, we performed genome-wide transcript profiling with *C. auris* biofilms following farnesol or tyrosol exposure using transcriptome sequencing (RNA-Seq). Since transition metals play a central role in fungal virulence and biofilm formation, levels of intracellular calcium, magnesium, and iron were determined following farnesol or tyrosol treatment using inductively coupled plasma optical emission spectrometry. Farnesol caused an 89.9% and 73.8% significant reduction in the calcium and magnesium content, respectively, whereas tyrosol resulted in 82.6%, 76.6%, and 81.2% decrease in the calcium, magnesium, and iron content, respectively, compared to the control. Genes involved in biofilm events, glycolysis, ergosterol biosynthesis, fatty acid oxidation, iron metabolism, and autophagy were primarily affected in treated cells. To prove ergosterol quorum-sensing molecule interactions, microdilution-based susceptibility testing was performed, where the complexation of farnesol, but not tyrosol, with ergosterol was impeded in the presence of exogenous ergosterol, resulting in a minimum inhibitory concentration increase in the quorum-sensing molecules. This study revealed several farnesol- and tyrosol-specific responses, which will contribute to the development of alternative therapies against *C. auris* biofilms.

**IMPORTANCE:**

*Candida auris* is a multidrug-resistant fungal pathogen, which is frequently associated with biofilm-related infections. *Candida*-derived quorum-sensing molecules (farnesol and tyrosol) play a pivotal role in the regulation of fungal morphogenesis and biofilm development. Furthermore, they may have remarkable anti-biofilm effects, especially at supraphysiological concentrations. Innovative therapeutic approaches interfering with quorum sensing may be a promising future strategy against *C. auris* biofilms; however, limited data are currently available concerning farnesol-induced and tyrosol-related molecular effects in *C. auris*. Here, we detected several genes involved in biofilm events, glycolysis, ergosterol biosynthesis, fatty acid oxidation, iron metabolism, and autophagy, which were primarily influenced following farnesol or tyrosol exposure. Moreover, calcium, magnesium, and iron homeostasis were also significantly affected. These results reveal those molecular and physiological events, which may support the development of novel therapeutic approaches against *C. auris* biofilms.

## INTRODUCTION

Since its first clinical description, *Candida auris* has emerged as a serious threat in the healthcare environment, warranting specific guidance by the Centers for Disease Control and Prevention and assignments to the critical group in the fungal priority pathogen list published recently by the World Health Organization (1, 2). Micafungin and amphotericin B have been recommended as the first-line therapy against *C. auris* for adults and infants, respectively; however, cases involving echinocandin-resistant isolates have tripled in the USA in the last 2 years (3–5). To further complicate therapy, indwelling medical devices were the sources of approximately 90% of *C. auris* candidemia cases, indicating that biofilm formation is one of the main predisposing factors for this invasive infection (6, 7). In addition, several data sets describe the development of resistance to echinocandins following the initial administration of these antifungals (8–10).

Quorum sensing is a well-known population density-based communication system through the release and sensing of different quorum-sensing molecules (11, 12). Farnesol and tyrosol are the two best-described quorum-sensing molecules in the case of *Candida* species. Under physiological conditions, farnesol inhibits the yeast-to-hyphal transition, while tyrosol has the opposite effect in terms of morphogenesis (13, 14). The observed inhibitory effect of these molecules at supraphysiological concentrations suggests that they could represent a potential part of innovative preventive strategies against *Candida* biofilms, including against the *C. auris* sessile community (15–19). Those studies showed that both molecules have a remarkable antifungal effect, interfering with redox homeostasis, virulence, and intracellular microelement contents against planktonic forms of *C. auris*; however, the transcriptome-based biofilm-related changes remain to be elucidated (17, 18, 20).

The present study reveals those molecular events, which may be associated with the previously observed antifungal effect exerted by these two quorum-sensing molecules. A detailed understanding of quorum-sensing molecule-related molecular mechanisms can drive the development of novel therapies to overcome this potentially multi-resistant fungal species.

## MATERIALS AND METHODS

### Isolate and culture conditions

*C. auris* isolate 12 (NCPF 8973), derived from the South Asian/Indian lineage, was obtained from the National Mycology Reference Laboratory (United Kingdom) (21). The strain was maintained on yeast extract–peptone–dextrose (YPD) solid medium [10 g/L of yeast extract (Alfa Aesar, USA), 20 g/L of mycological peptone (Oxoid, United Kingdom), 20 g/L of dextrose, and 20 g/L of agar (VWR International LLC, Hungary), pH 5.6]. Culturing and biofilm formation were performed in RPMI-1640 (with L-glutamine and without bicarbonate, pH 7.0, and with 3-(N-morpholino) propanesulfonic acid; Merck Ltd, Budapest, Hungary). Farnesol (Merck Ltd.) was obtained as a 3-M stock solution, which was diluted to 30 mM in 100% methanol. The working concentration of farnesol (75 µM) was prepared in the RPMI-1640 medium. Drug-free RPMI-1640 controls were supplemented with 1% (vol/vol) methanol. Tyrosol [2-(4-hydroxyphenyl) ethanol] (Merck Ltd.) was prepared as a 0.1-M stock solution in sterile physiological saline. The working concentration of tyrosol (15 mM) was prepared in RPMI-1640.

### Biofilm formation

Prior to biofilm-related experiments, *C. auris* isolate was subcultured on YPD agar for 48 h at 37°C. The whole *C. auris* culture from the solid agar was picked up using a common sterile swab and washed into 10 mL of sterile physiological saline. Suspensions were centrifuged at 3,000 × *g* for 5 min and washed three times with sterile physiological saline. Subsequently, pellets were re-suspended in physiological saline, and the cell density was adjusted to 1 × 10^6^ cells/mL in sterile RPMI-1640 media for each experiment using a Burker’s chamber (12, 14). A total of 550 µL of the adjusted *C. auris* suspension was placed in each well of 24-well polystyrene plates (TPP, Trasadingen, Switzerland) and supplemented with 450 µL of sterile RPMI-1640. Then plates were incubated statically for 24 h at 37°C. After the incubation time, the culture medium was aspirated, and non-adherent cells were removed by washing the biofilms with sterile physiological saline. Tyrosol and farnesol in 15 mM and 75 µM concentrations were added to preformed 1-day-old biofilms, and plates were incubated for a further 24 h at 37°C. Developed biofilms treated with farnesol or tyrosol were scraped from the 24-well plates, and the contents of corresponding wells (500 µL) were pooled together and then washed three times with physiological saline (15, 17, 18). Three biological replicates of biofilm-forming cell suspensions were centrifuged at 3,000 × *g* for 10 min at 4°C, and the pellets were used for RNA extraction and intracellular metal content measure. Biofilm growth was characterized by measurement of dry cell mass (DCM) after freeze-drying of the biomass.

### RNA extraction

Total RNA samples were isolated from lyophilized *C. auris* cells (CHRIST Alpha 1-2 LD plus lyophilizer, Osterode, Germany) using Tri Reagent (Merck Ltd.) exactly as described in our previous studies (18, 20).

### Reverse-transcription quantitative real-time PCR (RT-qPCR) assays

For RT-qPCR, 1  µg of total RNA from each of three independent experiments was digested with DNase I (Merck Ltd.) following the manufacturer’s instructions, and the expression levels of genes were quantified with the Luna Universal One-Step RT-qPCR Kit (New England BioLabs, Ipswich, MA, USA) with the following cycling parameters: 10 min at 55°C and 1  min at 95°C, followed by 40 cycles of 10  s at 95°C, 10  s at 51°C, and 20  s at 65°C. The relative expression of each gene was normalized to that of the *ACT1* (B9J08_000486) gene. Oligonucleotide primers were designed with Oligo Explorer (v.1.1.) (https://oligo-explorer.software.informer.com/1.1/) and Oligo Analyzer (v.1.0.2) (https://oligo-analyzer.software.informer.com) software and are listed in Table S1. Relative transcription levels were quantified with the ΔΔCP method using the formula ΔΔCP = ΔCP_control_ − ΔCP_treated_, where ΔCP_control_ = CP_tested gene_ − CP_reference gene_, measured from control cultures, and ΔCP_treated_ = CP_tested gene_ − CP_reference gene_, measured from treated cultures (18, 20). CP values represent qRT-PCR cycle numbers of crossing points.

### RNA sequencing

Total RNA was isolated from the farnesol-treated, tyrosol-treated, and untreated biofilms of *C. auris* isolate 12. Whole RNA sequencing from ∼250 ng of high-quality total RNA was performed at the Genomic Medicine and Bioinformatic Core Facility, Department of Biochemistry and Molecular Biology, Faculty of Medicine, University of Debrecen, Debrecen, Hungary. To evaluate the total RNA sample quality, an Agilent BioAnalyzer was employed using the Eukaryotic Total RNA Nano Kit (Agilent Technologies, Inc., Santa Clara, CA, USA) as described in the manufacturer’s protocol (18, 20). Library preparation was performed from samples with an RNA integrity number higher than 7. Libraries were prepared with the NEBNext RNA Sample Preparation kit (New England BioLabs) according to the manufacturer’s protocol. Biofilm samples were sequenced (single-read 75-bp sequencing) on an Illumina NextSeq 500 instrument (Illumina, San Diego, CA, USA) separately. Depending on the sample type, 19–23 million reads per sample (farnesol treated, tyrosol treated, or untreated samples) were obtained. The FastQC package (www.bioinformatics.babraham.ac.uk/projects/) was used for quality control. Reads were aligned to the genome of *C. auris* B8441, retrieved from the Candida Genome Database (CGD) (www.candidagenome.org) with the HISAT2 algorithm combined with SAMtools (22). The successfully aligned reads of three experimental settings varied between 92% and 100%.

Regarding HISAT parameters, our fastp reports showed that a portion of the low-quality reads was approximately 2% in our raw data files. Adapter sequences were detected in only three samples, where three portions were 0.093%, 0.0927%, and 0.115%. The Q30 bases are >89% in the whole data set. These quality control results suggest that the raw data have good quality. Moreover, the fastp generation method of Illumina automatically does the trimming and removing of adapter sequences from reads when the bcl does the conversion to fastp. HISAT2 was used with the default parameters; these settings are available at the following website: http://daehwankimlab.github.io/hisat2/. The default settings of HISAT2 are suitable for removing adapter sequences and low-quality reads.

For the downstream analysis, we used the StrandNGS software, which is a next-generation sequencing data analysis tool. It supports the analysis of different types of NGS data, such as DNA-Seq, RNA-Seq, CHIP-Seq, Methyl-Seq, and small RNA-Seq. Raw (FASTQ, FASTA) and pre-aligned (BAM, SAM) data can be imported for analysis. This is a user-friendly software with a graphical interface for those researchers who do not have a bioinformatic background or the help of a bioinformatic team. It contains workflow for the analysis and visualization of RNA-Seq data including standard differential expression analysis for different experimental conditions (https://www.strand-ngs.com/files/manual/reference/rnaseq.html#SECTION00420000000000000000, https://www.strand-ngs.com/files/highlights/RNA-seq.pdf).

The workflow contains the following steps:

1. Quantification—generation expression values at gene, exon, and transcript levels.
2. Normalization—generation-normalized expression values; different algorithms are available such as DESeq, RPM, TMM, and quantile methods.
3. Statistical test for determining differential expression: *t*-tests, Mann–Whitney, and analysis of variance for identifying differentially expressed genes under different experimental conditions.
4. Multiple testing correction using Benjamini–Hochberg, Storey, Bonferroni, etc.

Regarding expression value calculation, we decided to import the pre-aligned BAM file format. During the analysis, we followed the default RNA-Seq workflow. The software-integrated DESeq algorithm was used for generating normalized gene expression values, and these values were used for determining the differentially expressed genes between the farnesol- and tyrosol-treated samples by moderated *t*-test with Benjamini–Hochberg false discovery rate for multiple testing correction.

### Evaluation of transcriptome data

The CGD platform (www.candidagenome.org) with default settings [function, process, and component gene ontology (GO) terms)] was used to characterize the up- and downregulated differentially expressed gene sets. Only hits with a corrected *P* value <0.05 were regarded as significantly enriched (Table S2).

Enrichment of selected genes belonging to the “virulence-associated genes,” “metabolic pathway-associated genes,” “iron metabolism-associated genes,” and “autophagy-related genes” groups in the up- and downregulated gene sets was studied with the Fisher’s exact test function of the R project (www.R-project.org/) (Table S3). The “virulence-associated genes” are known as putative genes involved in the genetic regulation of *Candida albicans* virulence properties (morphogenesis, adhesion, invasion, biofilm formation, and putative ABC transporters) according to previously published classifications (23–25). The “metabolic pathway-associated genes” include all genes related to ergosterol, carbohydrate, and fatty acid biochemical pathways based on the pathway databases (http://pathway.candidagenome.org/). The “iron metabolism-associated genes” were collected by the method proposed by Fourie et al. (26) and Gerwien et al. (27) (26, 27). “Autophagy-related genes” were collected from the CGD (www.candidagenome.org).

### Intracellular metal contents measured by inductively coupled plasma optical emission spectrometry in *Candida auris* biofilms

The selected intracellular element (Fe, Ca, and Mg) contents of the lyophilized biomass were determined by inductively coupled plasma optical emission spectrometry (5110 Agilent Technologies) following atmospheric wet digestion in 3 mL of 65% HNO_3_ and 1 mL of 30% H_2_O_2_ in glass beakers. The metal contents of the samples were normalized by DCM as described by Jakab et al. (20). The metal contents of the dry biomass were determined in triplicate, and mean ± standard deviation values were presented.

### Ergosterol-binding assay

To determine the binding of farnesol or tyrosol to the ergosterol present in *C. auris* cell membranes, an ergosterol-binding assay was performed on planktonic cells as described by Ramesh et al. (28). Briefly, ergosterol (Merck) was dissolved in dimethyl sulfoxide and then applied and diluted to 100- and 200-mg/L final concentrations in RPMI-1640. The minimum inhibitory concentration (MIC) values of farnesol or tyrosol against *C. auris* were determined in RPMI-1640 according to the recommendations proposed by the Clinical Laboratory Standards Institute M27-A3 protocol with and without media supplemented with ergosterol (29). The concentrations tested ranged from 0.585 to 300 µM for farnesol and from 0.058 to 30 mM for tyrosol, with 100 and 200 mg/L of ergosterol in RPMI-1640. MICs were determined as the lowest concentration that caused at least 50% growth inhibition compared to the untreated control cells. The changes in MIC values with and without added ergosterol were determined to allow calculation of the ergosterol-binding ability of farnesol and tyrosol.

## RESULTS

### Genome-wide transcriptional changes for *Candida auris* biofilms

**Fig 1:**
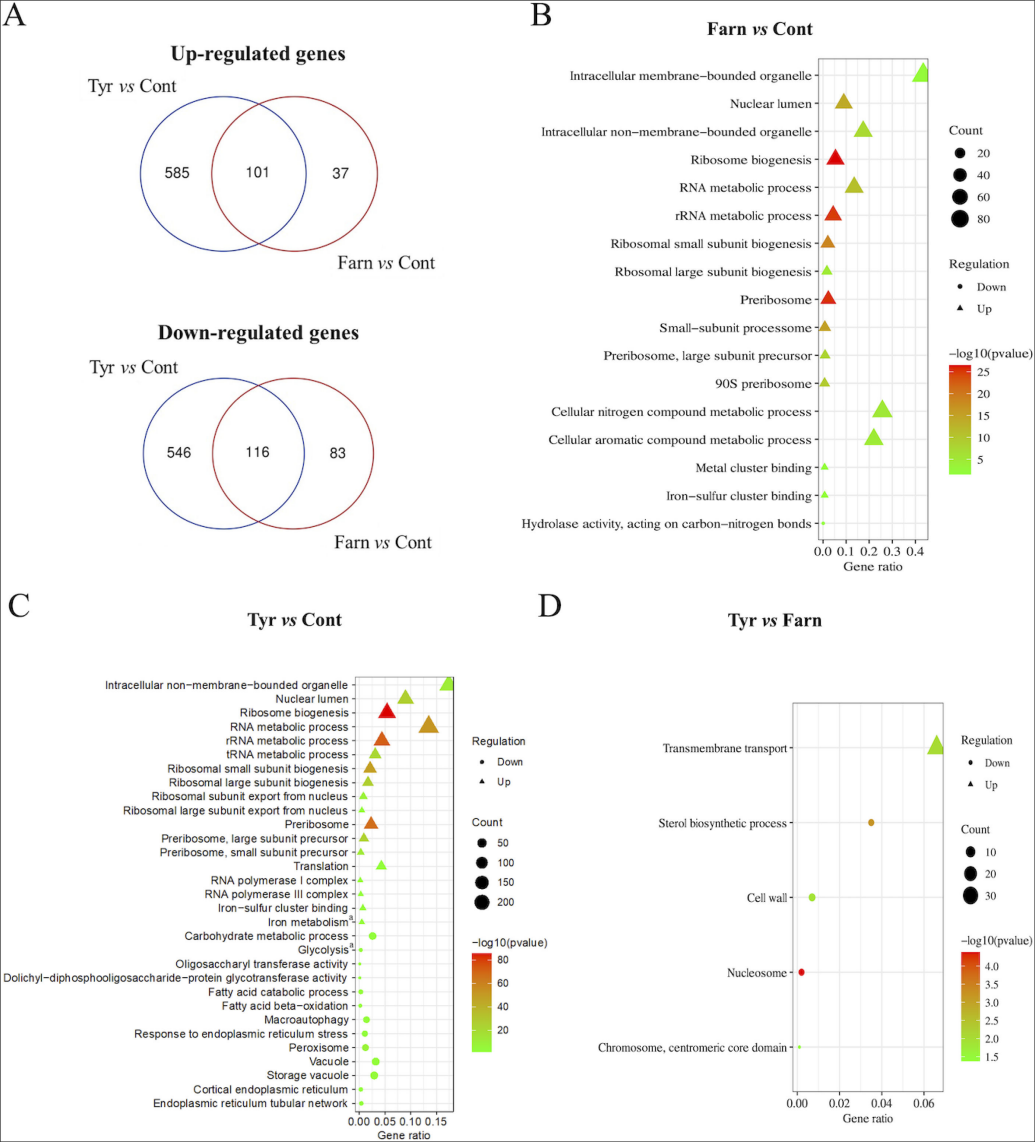
Summary of RNA-Seq data and main gene enrichment analyses. (**A**) The effects of tyrosol (Tyr vs Cont) and farnesol (Farn vs Cont) treatment on the transcriptomes are depicted in the Venn diagrams. (**B–D**) Bubble charts of GO terms of CGD (http://www.candidagenome.org/cgi-bin/GO/goTermFinder) and results of the Fisher’s exact test generated by different expression genes. Bubble charts represent up- (Δ) and downregulated (•) genes belonging to gene groups farnesol-treated vs untreated (**B**), tyrosol-treated vs untreated (**C**), and farnesol- vs tyrosol-treated (**D**) comparisons where the enrichment was significant (*P* < 0.05). The color of the bubble means the significance of the corresponding pathway (in green color, low *P* values; in red color, high *P* values). In addition, the size of the bubble means the number of different expression genes in this pathway. Only the differentially expressed genes (corrected *P* value of <0.05) exhibiting more than a 1.5-fold increase or decrease in their transcription are shown. The full list of the significantly enriched GO terms is available in Table S2. Significant enrichment in the appropriate gene set according to Fisher’s exact test (*P* < 0.05). The full data set is available in Table S3.

**Fig 2:**
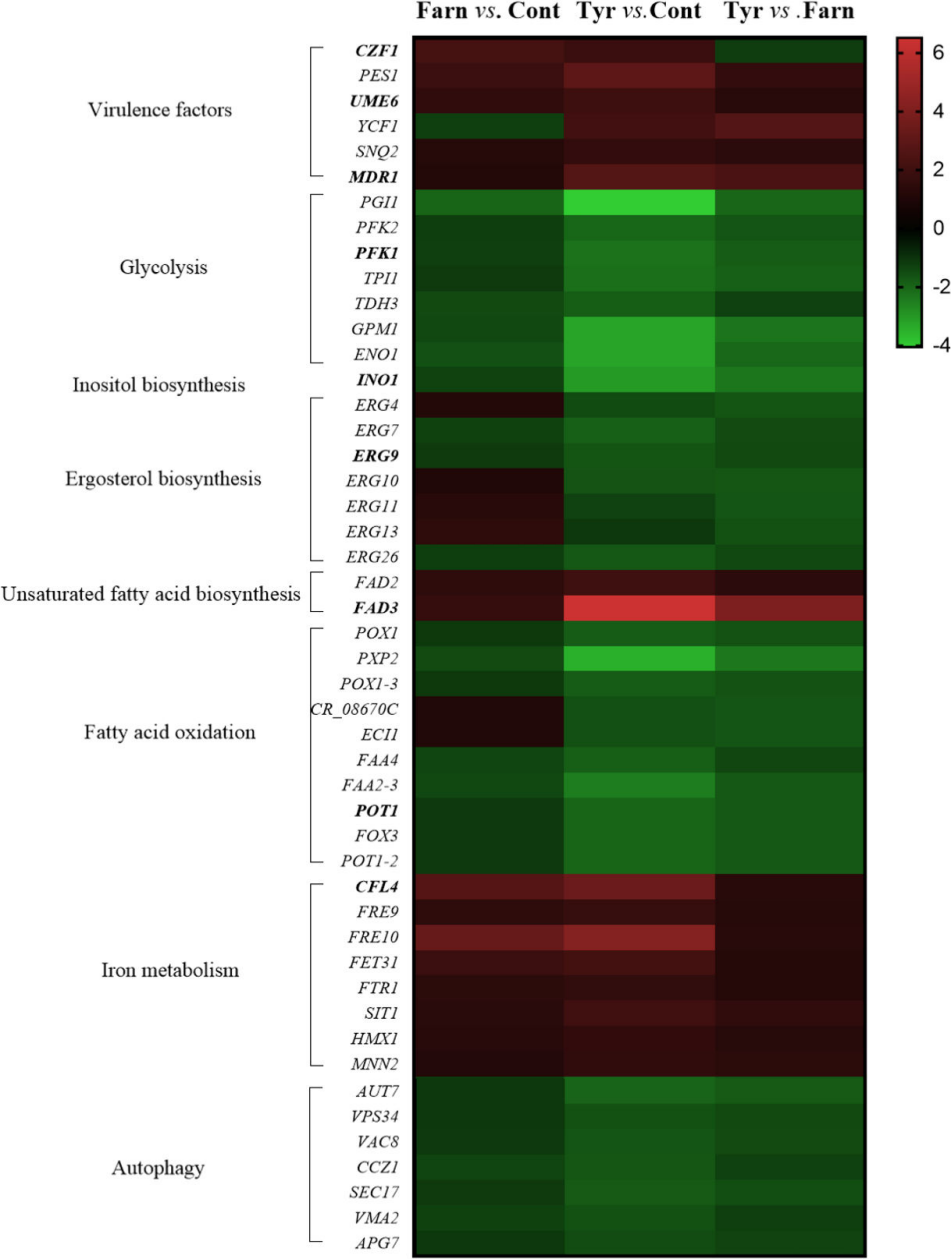
The effects of quorum-sensing compounds, farnesol and tyrosol, on the expression of selected genes of *Candida auris* biofilms. The heat map demonstrates the expression profiles of representative genes according to the color scale that indicates gene expression changes in fold change units. Table S3 summarizes the data that were used for the construction of the heat map. Bold names indicate the genes that were selected for the RT-qPCR analysis. The data set for genes is available in Table S4 in the supplemental material.

Reproducible relationships between RNA-Seq results were confirmed by principal component analysis (Fig. S1). Furthermore, the effects of quorum-sensing molecules on the transcriptomes are summarized in Fig. 1A through D and Fig. 2.

Tyrosol-related effects on *C. auris* biofilms were more pronounced than the farnesol-related effects when compared with untreated control sessile cells. The number of upregulated genes was 686 and 138 for tyrosol and farnesol, respectively, while 662 and 199 genes were downregulated for tyrosol and farnesol, respectively (Fig. 1A). The overlaps between tyrosol- and farnesol-responsive genes were considerable (101 and 116 overlapping up- and downregulated genes, respectively); however, the transcription of several genes changed exclusively in response to tyrosol exposure (the number of up- and downregulated tyrosol-responsive genes was 585 and 546, respectively (Fig. 1A).

RT-qPCR was performed to quantify the transcription of 11 selected genes (six upregulated genes: *UME6*, *CFL4*, *BIO2*, *CZF*1, *FAD3*, and *MDR1*; three downregulated genes: *PFK1*, *INO1*, and *POT1*; and two non-differentially expressed genes: *ACT1* and *ERG9*) selected on the basis of the RNA-Seq experiments (Table S1). The fold change obtained using RNA-Seq was compared with relative transcription levels (ΔΔCP) derived from RT-qPCR analysis. The similarity between the transcription levels obtained from the two methods indicates high consistency between the analytical data. Table S4 indicates a good correlation between RNA-Seq and RT-qPCR data with a correlation coefficient (*r*) of 0.89 (farnesol vs control) and 0.95 (tyrosol vs control). Transcriptional changes for up- and downregulated genes were further characterized using gene set enrichment analyses (Fig. 1B through D; Tables S2 and S3), and selected changes are illustrated in a heat map (Fig. 2).

### Farnesol-associated transcriptomic effect in *Candida auris* biofilm

Based on our transcriptomic data, genes involved in biofilm formation (*CZF1*, *UME6*, and *TYE7* transcription factors; and *PES1*, encoding a key enzyme specific to the regulation of the hyphae-to-yeast transition), iron-sulfur cluster binding (*RLI1*, *ISA1*, *BIO2*, *SDH2*, *DRE2*, and *LEU1*), iron uptake (*FET31*, multicopper oxidase; *CCC1*, ferrous iron transporter; *CFL4* and *FRE10*, ferric reductases), and ribosome biogenesis (50 genes), ribosomal small subunit biogenesis (29 genes), ribosomal large subunit biogenesis (14 genes), RNA metabolic process (54 genes), and rRNA metabolic process (45 genes) were enriched in the upregulated gene set (Fig. 1 and 2; Tables S2 and S3). Upregulation of *UME6*, *CZF1*, *BIO2*, and *CFL4* under farnesol exposure was confirmed by RT-qPCR data (Table S4).

### Tyrosol-associated transcriptomic effect in *Candida auris* biofilm

Transcripts of biofilm-formation genes (*CZF1*, *UME6*, and *TYE7* transcription factors; and *PES1*) were also activated by tyrosol treatment (Fig. 2; Table S3). Furthermore, significant upregulation was observed in the case of the following genes: putative ABC transporters (*MDR1*, *YCF1*, and *SNQ2*) and unsaturated fatty acid biosynthetic process (*FAD2* and *FAD*3 encoding for delta-12 and omega-3 fatty acid desaturases) (Fig. 2; Table S3). In addition, tyrosol treatment resulted in the enrichment of upregulated iron homeostasis (*CFL4*, *FRE9*, and *FRE10*, ferric reductases; *FET31*, multicopper oxidase; *FTR1*, iron permease; *SIT1*, ferrichrome siderophores transporter; *HXM1*, heme oxygenase; *MNN2* and *CCC1*, iron transporters), iron-sulfur cluster binding (15 genes, e.g., *RLI1*, *ECM17*, *YAH1*, *ISA1*, *LYS4*, *BIO2*, *ELP3*, *SDH2*, *DRE2*, and *LEU1*), and ribosome biogenesis (175 genes), ribosomal small subunit biogenesis (84 genes), ribosomal large subunit biogenesis (57 genes), RNA metabolic process (241 genes), rRNA metabolic process (147 genes), tRNA metabolic process (74 genes), RNA polymerase I complex (8 genes), RNA polymerase III complex (9 genes), and translation (52 genes) (Fig. 1 and 2; Tables S2 and S3).

Conversely, ergosterol biosynthetic process (*ERG4*, *ERG7*, *ERG9*, *ERG10*, and *ERG26*), phospholipid binding (26 genes), carbohydrate metabolic process [36 genes, e.g., inositol metabolic process (*INO1* and CR_08,330W), trehalose metabolism (*TPS1*), glycolysis (*PGI1*, *PFK2*, *PFK1*, *TPI1*, *TDH3*, *GPM1*, and *ENO1*) and maltose degradation (*MAL2*, C5_04,940W, *GDB1*, and C5_04940W9)], fatty acid metabolic process [20 genes, e.g., fatty acid beta-oxidation (*POX1*, *PXP2*, *POX1-3*, CR_08,670C, *ECI1*, *FAA4*, and *FAA2-3*) and glyoxylate cycle (*MLS1* and *MDH1-3*)], carboxylic acid catabolic process [22 genes, e.g., glutamate degradation (*GAD1*, *UGA11*, and *UGA2*)], macroautophagy (25 genes), and response to endoplasmic reticulum stress (20 genes) were enriched in the downregulated gene set (Fig. 1 and 2; Tables S2 and S3).

Moreover, tyrosol exposure significantly decreased the transcription of 28 peroxisomal genes, 42 vacuolar genes, 37 genes of the cell cortex, including 9 genes of the cortical endoplasmic reticulum and 20 genes of the cortical actin cytoskeleton, and 9 genes of the endoplasmic reticulum tubular network in the cellular component-related gene set (Fig. 1; Table S2). It is noteworthy that tyrosol treatment caused a significant increase in the transcription of *UME6*, *CZF1*, *FAD3*, *BIO2*, *CFL4*, and *MDR1* based on the RT-qPCR measurements. In addition, the downregulation of *PFK1*, *INO1*, and *POT1* was also supported by RT-qPCR (Table S4).

The obtained data indicated that tyrosol exposure significantly increased the transcription of 30 transmembrane transport-related genes and decreased the expression of four ergosterol biosynthetic process (*ERG4*, *ERG10*, *ERG11*, and *ERG13*)-related genes compared to farnesol treatment (Fig. 1 and 2; Tables S2 and S3).

### Quorum-sensing molecules significantly influence the metal contents of 1-day-old *Candida auris* biofilm

Transition metals provide a considerable role as cofactors for different enzymes in virulence and in biofilm formation (27). Farnesol and tyrosol exposures significantly influence the calcium (319.37 ± 234.80 and 551.75 ± 441.83 mg/kg) and magnesium (695.78 ± 111.91 and 618.65 ± 40.75 mg/kg) contents of *C. auris* biofilms compared to controls (3170.7 ± 82.8 mg/kg for calcium and 2648.36 ± 35.05 mg/kg for magnesium, respectively) (Table 1). Although both the tested molecules led to decreases in intracellular iron content, this reduction was not statistically significant in the case of farnesol compared to untreated control (240.34 ± 118.39, 67.17 ± 15.84, and 356.32 ± 45.62 mg/kg for farnesol, tyrosol, and control, respectively) (Table 1). In addition, a significant decrease was detected in the DCM of farnesol- and tyrosol-treated biofilms (0.53 ± 0.165 and 0.4 ± 0.16 g/L for farnesol and tyrosol, respectively) compared to untreated cells (1.37 ± 0.35 g/L) (Table 1).

**TABLE 1:** Effects of quorum-sensing molecules significantly influence the metal contents of *Candida auris* biofilms^*b*^

Culture	DCM(g/L)	Metal contents/treatment (mg/kg) (mean ± SD^*a*^)		
		Ca	Mg	Fe
Control cultures	1.37 ± 0.35	3,170.7 ± 82.8	2,648.36 ± 35.05	356.32 ± 45.62
+75 µM farnesol	0.53 ± 0.165**	319.37 ± 234.80**	695.78 ± 111.91**	240.34 ± 118.39
+15 mM tyrosol	0.4 ± 0.16**	551.75 ± 441.83**	618.65 ± 40.75**	67.17 ± 15.84**

^*a*^ Mean values ± SD calculated from three independent experiments are presented.

^*b*^ The asterisks indicate significant differences calculated by Student’s *t*-test comparing untreated control and farnesol or tyrosol-treated cultures as follows: **P< 0.01.

### Ergosterol-binding assay

The ability of farnesol or tyrosol to cause membrane destabilization can be inferred by its ability to interfere with exogenous ergosterol added to the planktonic *C. auris* suspension in a standard microdilution assay. In the presence of exogenous ergosterol at 100 and 200  mg/L, the MIC of farnesol increased fourfold, from 75 to 300 µM for *C. auris*. In the combination of tyrosol and ergosterol, the MIC values were 30 mM in the presence or absence of ergosterol. These results indicate that farnesol, but not tyrosol, may exert its activity in whole or in part by binding to membrane ergosterol.

## DISCUSSION

Previous studies showed that anti-biofilm strategies interfering with quorum sensing may effectively target *C. auris* biofilms (30–32). Both farnesol and tyrosol, especially at supraphysiological concentrations, have remarkable antifungal and drug potentiator effects against several *Candida* species including *C. auris* (15–20). It is noteworthy that previously performed differential expression analysis demonstrated that the *C. auris* planktonic and biofilm transcriptome differ significantly (33). Therefore, the effects of quorum-sensing molecules on planktonic cell findings could not be directly extrapolated to biofilms.

Our comparative transcriptomic data show a significant upregulation in *CZF1* and *UME6* genes following both farnesol and tyrosol exposure. A similar upregulation was observed for *TYE7*, which is the major transcriptional regulator of glycolysis genes in *C. albicans* that binds the promoters of genes related to glycolysis such as *PFK1-* and *PFK2*-encoding subunits of phosphofructokinase (34). This enzyme irreversibly converts fructose-6-phosphate into fructose-1,6-bisphosphate, which is a pivotal regulatory step in glycolysis (34, 35). Furthermore, it acts as a negative regulator of hypoxic filamentation (36). Despite the overexpression of *TYE7*, several key genes in glycolysis were significantly downregulated (*PGI1*, *PFK1*, *PFK2*, *TPI1*, *TDH3*, *GPM1*, and *ENO1*), especially under tyrosol exposure. The opposite pattern was reported in *Candida parapsilosis* planktonic cells, where exogenous tyrosol treatment shifted metabolism toward glycolysis (18). Overexpression of Czf1 protein stimulates filamentation; moreover, *CZF1* gene deletion is associated with negative effects on hyphae filamentation. A similar *CZF1* upregulation was observed in the case of *C. parapsilosis* planktonic cells following tyrosol exposure; however, Jakab et al. (18) did not observe higher rates of adherence and biofilm-forming ability in the presence of this quorum-sensing molecule (18). The gene of *UME6* is also important for hyphal extension. In addition, Ume6 protein plays a pivotal role in the expression of *HWP1*, *ECE1*, *ALS3*, and *HCG1*, which are associated with filamentation (35, 36). We hypothesize that the observed upregulation of *CZF1* and *UME6* is a compensatory response of fungi to maintain the biofilm structure because both farnesol and tyrosol exposure significantly decreased the level of two bivalent cations—magnesium and calcium—which play a critical role in biofilm development (37–41).

Previous studies suggest that magnesium triggers the growth of filamentous forms in *C. albicans* and in *Trichosporon asahii* (37, 38). Furthermore, magnesium uptake has an effect on mitochondrial distribution, the production of lipid droplets, and vacuolar growth, which contribute to the promotion of hyphal growth and directly to biofilm formation (38). In our experiments, intracellular magnesium level was decreased, which can influence the number of physiological effects. Hans et al. (39) showed that magnesium deprivation impedes the metabolic flexibility of *C. albicans* (39). In our study, several glycolysis-, gluconeogenesis-, and fatty acid oxidation-related genes were downregulated, especially after tyrosol treatment, which were associated with the reduced growth rate and the significantly decreased dry cell mass of sessile cells. The decreased magnesium content inhibited potential virulence traits, including biofilm formation, morphological transition, and adherence to epithelial cells; moreover, it significantly influences membrane homeostasis with remarkable changes in ergosterol synthesis-related genes, as confirmed in this study (39). A further study revealed that lower magnesium concentration led to the potentiation of membrane-targeting antifungal drugs, which was confirmed previously for farnesol and the triazoles against *C. auris* biofilms (17, 39).

In addition to the effects on magnesium content, both farnesol- and tyrosol-treated biofilms showed a decreased calcium content. Previous results demonstrated that calcium supplementation could increase the length of fungal cells grown for *T. ashaii*, *Cryptococcus neoformans*, and *C. albicans* because calcium regulates both actin polymerization and microtubule polymerization; thus, it has a remarkable direct effect on biofilm development (40, 41). In accordance with these studies and alongside the decreased calcium levels, tyrosol treatment significantly downregulated the transcription of several genes, which influence the actin filament organization, actin cortical patch, cortical cytoskeleton, and cortical actin cytoskeleton. Presumably, the simultaneous reduction of these two crucial bivalent cations may explain the previously documented anti-biofilm effect exerted by farnesol or tyrosol.

Tyrosol treatment significantly decreased the iron content of biofilms, in association with several upregulated iron homeostasis-related gene groups (e.g., ferric reductases, multicopper oxidases, and iron permeases). Although farnesol exposure resulted in a similar pattern in the transcription level of these genes, the observed changes did not coincide with significantly decreased iron content. Nevertheless, previously published planktonic *C. auris* transcriptomic data showed that farnesol treatment downregulated the transcription of iron homeostasis-related genes, which were associated with a significant reduction in iron concentration (20). It is noteworthy that iron deprivation does not influence the biofilm-forming ability of *C. albicans* (42). Nonetheless, the decreased iron content enhances the membrane fluidity of *Candida* cells, influencing their susceptibility to membrane-active antifungal agents (43).

Considering the results derived from transcriptome analysis, intracellular metal content determination, and ergosterol-binding assay, the examined fungal quorum-sensing molecules appear to impact the fungal cell membrane structure. Our planktonic cell-based ergosterol-binding assay shows that farnesol is highly bound to ergosterol, which presumably changes the conformational properties of ergosterol, influencing the membrane characteristics. Further structure-based confirmatory experiments are needed to test this hypothesis, especially in the case of biofilms where extracellular matrix may also influence diffusion properties. Tyrosol could also influence certain membrane characteristics. Tyrosol treatment significantly enhanced the transcription of *FAD2* and *FAD3* genes encoding for fatty acid desaturases involved in polyunsaturated fatty acid synthesis. Riekhof et al. (44) demonstrated a similar pattern in *FAD2*/*FAD3* transcription following phosphate starvation in fungi (44). The overexpression of these desaturases may increase the tolerance of fungal cells to environmental stress.

Another remarkable tyrosol-induced membrane-related effect was the downregulation of several ergosterol synthesis-associated genes, including *ERG4*, *ERG7*, *ERG9*, *ERG10*, and *ERG26*. The downregulation of these genes may alter membrane permeability and influence its fluidity. For farnesol, Dižová et al. showed that farnesol exposure (200 µM) downregulated the *ERG9*, *ERG11*, and *ERG20* genes in *C. albicans* (45). Furthermore, Jakab et al. (20) reported that the presence of 75 µM of farnesol decreases the transcription of *ERG6* gene in *C. auris*, which might enhance the passive diffusion of farnesol. Additionally, the resulting decreased *ERG6* content increases the susceptibility to oxidative stress and impairs thermotolerance (20). Surprisingly, farnesol did not cause any relevant change in the transcription of central ergosterol biosynthesis-related genes in this study. Aside from *ERG* genes, *INO1*, encoding inositol-1-phosphate synthase, was also downregulated following tyrosol exposure. Interestingly, in the case of planktonic *C. auris* cells, farnesol reduces the transcription of this gene (20).

With respect to autophagy-related genes, tyrosol exposure caused a significant decrease in the transcription of *C1_00,430W*, *AUT7*, *VPS34*, *C4_01,790W*, *VAC8*, *CCZ1*, *C7_03,860W*, *SEC17*, *VMA2*, and *APG7*, whereas the transcription level of *SPO72* was increased. Macroautophagy is an evolutionarily conserved dynamic pathway that functions primarily in a degradative manner. Macroautophagy has a pivotal role in the maintenance of cellular homeostasis; however, either under- or overactivated macroautophagy can remarkably compromise cell physiology, leading to cell death (46).

This is the very first study analyzing the global changes in gene transcription of *C. auris* biofilms in a comparative manner following farnesol and tyrosol exposure. However, two limitations should be highlighted. First, the timing of the quorum-sensing molecule exposure influences the physiological and transcriptome effects (13, 17). For example, farnesol has no significant effect on *C. albicans* cells that have already begun hyphal or biofilm development at least at physiological concentrations (13). However, remarkable species-specific differences are observed in the case of *C. auris* or *C. parapsilosis* compared to *C. albicans* (13, 19). Different farnesol concentrations inhibited the *C. auris* cells during early, but not late, biofilm-forming events. In contrast, the same tested concentrations inhibited the *C. albicans* cells compared to untreated control at 24 h (13). Indeed, farnesol exerted a potent anti-biofilm effect against *C. auris*, but not against *C. albicans,* compared to the control when tested on 1-day-old biofilms of each (13). Second, tyrosol was diluted only in sterile physiological saline without methanol, and it is possible that genes that change under tyrosol exposure are due to the comparison of culture with methanol. Nevertheless, Yasokawa et al. (47) showed that methanol exposure at lower concentrations (1.23 M) does not influence the growth of another yeast, *Saccharomyces cerevisiae*; furthermore, differences in gene transcription were not detected compared to the untreated control cells (47). Mota et al. (48) compared the susceptibility of *S. cerevisiae* to methanol [0%–14% (vol/vol)] using growth curve analysis and spot assays. The 5% (vol/vol) methanol treatment did not significantly affect the growth pattern of yeast at the first 36 h. Moreover, methanol concentrations ranging from 8% to 10% (vol/vol) did not show differences in the growth of treated fungal cells at 24 h compared to that of the control. The published results of Yasokawa et al. (47) and Mota et al. (48) suggest that oxidative stress is one of the major consequences of methanol exposure [1.23 M or 8% (vol/vol)]. Regarding our transcriptomic data, tyrosol exposure did not influence the transcription of oxidative stress-responsive genes and the several alcohol dehydrogenase- and repair system-related genes—which may be influenced primarily by methanol—compared to farnesol-treated or 1% (vol/vol) methanol-treated control cells.

Although our data give several potential physiological and molecular explanations for the previously observed quorum-sensing molecule-related antifungal effects, further mutant-based *in vitro* and *in vivo* investigations are needed to fully understand the complete mechanisms of farnesol and tyrosol action in the *C. auris* sessile community.

## Data Availability

Transcriptome data have been deposited in NCBI’s Gene Expression Omnibus (GEO) and are accessible through GEO Series accession number GSE233427.

## SUPPLEMENTAL MATERIAL

Figure S1 spectrum.02278-23-s0001.pdf Principal component analysis of RNAseq data.

Table S1 spectrum.02278-23-s0002.xlsx Oligonucleotide primers used for RT-qPCR analysis.

Table S2 spectrum.02278-23-s0003.xlsx Results of the gene set enrichment analysis.

Table S3 spectrum.02278-23-s0004.xlsx Transcription data of selected gene groups.

Table S4 spectrum.02278-23-s0005.xlsx Overview of RT-qPCR assays.

ASM does not own the copyrights to Supplemental Material that may be linked to, or accessed through, an article. The authors have granted ASM a non-exclusive, world-wide license to publish the Supplemental Material files. Please contact the corresponding author directly for reuse.
