# Supplementary figures and images for: Comparative transcriptional analysis of Candida auris biofilms following farnesol and tyrosol treatment
Source: Microbiol Spectr. 2024 Mar 5;12(4):e02278-23. doi: 10.1128/spectrum.02278-23 (PMC10986546; doi:10.1128/spectrum.02278-23)

Figure S1 Principal component analysis of RNAseq data

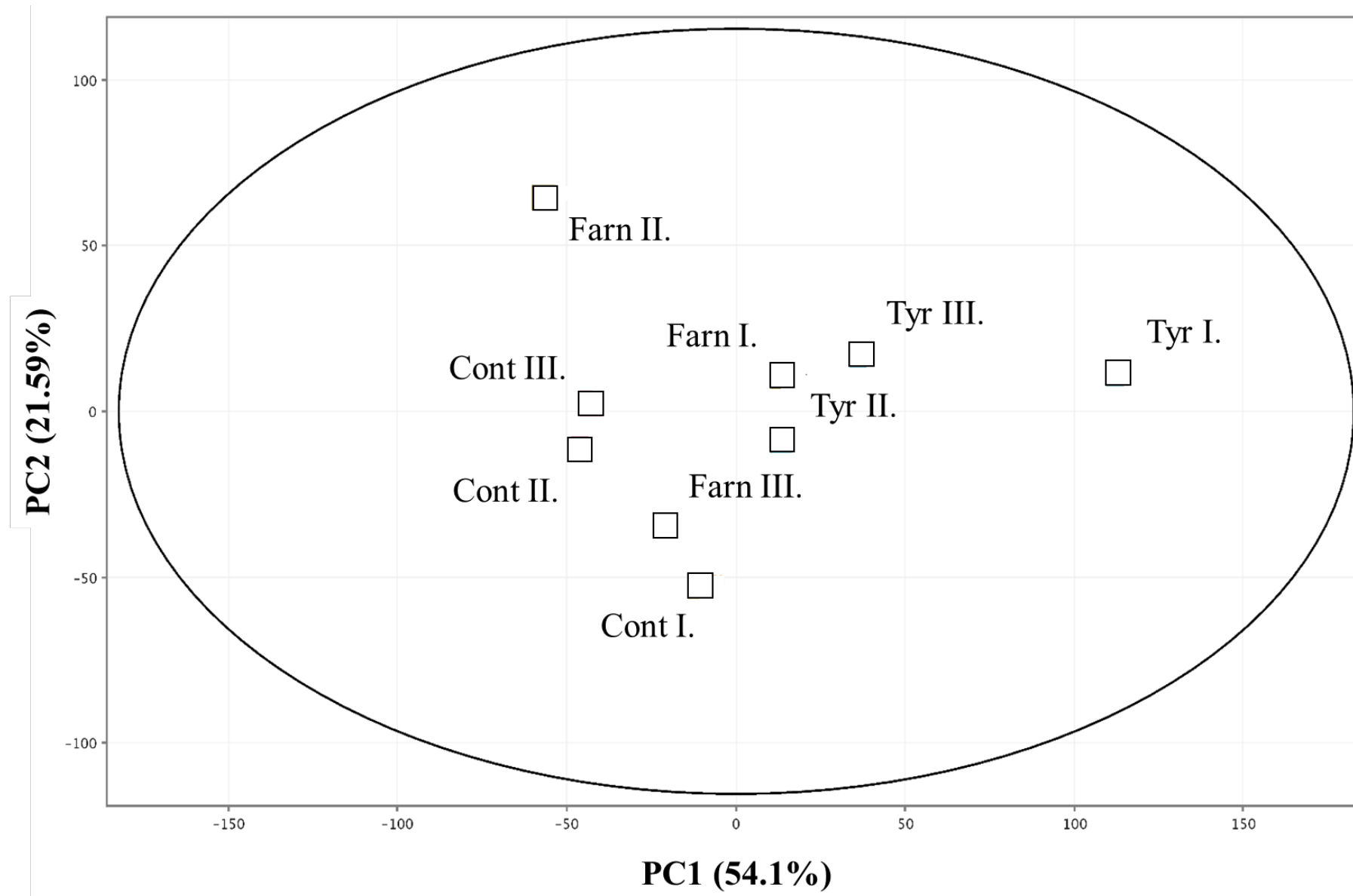

Supplement: Figure S1 — Principal component analysis of RNAseq data. [file spectrum.02278-23-s0001.pdf]
